# CD99 at the crossroads of physiology and pathology

**DOI:** 10.1007/s12079-017-0445-z

**Published:** 2018-01-06

**Authors:** Michela Pasello, Maria Cristina Manara, Katia Scotlandi

**Affiliations:** Experimental Oncology Lab, CRS Development of Biomolecular Therapies, Orthopaedic Rizzoli Institute, via di Barbiano 1/10, 40136 Bologna, Italy

**Keywords:** CD99, Cell migration, Cell differentiation, Metastasis, Cellular signaling

## Abstract

CD99 is a cell surface protein with unique features and only partly defined mechanisms of action. This molecule is involved in crucial biological processes, including cell adhesion, migration, death, differentiation and diapedesis, and it influences processes associated with inflammation, immune responses and cancer. CD99 is frequently overexpressed in many types of tumors, particularly pediatric tumors including Ewing sarcoma and specific subtypes of leukemia. Engagement of CD99 induces the death of malignant cells through non-conventional mechanisms. In Ewing sarcoma, triggering of CD99 by specific monoclonal antibodies activates hyperstimulation of micropinocytosis and leads to cancer cells killing through a caspase-independent, non-apoptotic pathway resembling methuosis. This process is characterized by extreme accumulation of vacuoles in the cytoplasmic space, which compromises cell viability, requires the activation of RAS-Rac1 downstream signaling and appears to be rather specific for tumor cells. In addition, anti-CD99 monoclonal antibodies exhibit antitumor activities in xenografts in the absence of immune effector cells or complement proteins. Overall, these data establish CD99 as a new opportunity to treat patients with high expression of CD99, particularly those that are resistant to canonical apoptosis-inducing agents.

## Introduction

CD99 is a transmembrane molecule that is encoded by the pseudoautosomal gene *MIC2* (Goodfellow et al. 1988). This molecule is highly O-glycosylated and together with Xga and CD99 antigen-like 2 (CD99L2) constitutes a family of molecules that show no homology to any other known family (Ellis et al. 1994a; Suh et al. 2003; Tippett and Ellis 1998)*.*

CD99 is broadly expressed in humans and primates. Although ubiquitously expressed in almost all human cell types at low levels, CD99 displays strong expression in a particular subtypes of cells (see below for details) and it is involved in essential functions including apoptosis (Bernard et al. 1997; Cerisano et al. 2004; Husak et al. 2010; Jung et al. 2003; Pettersen et al. 2001; Sohn et al. 1998), adhesion (Bernard et al. 1995, 2000; Cerisano et al. 2004; Hahn et al. 1997; Kasinrerk et al. 2000), differentiation (Huang et al. 2012; Rocchi et al. 2010; Sciandra et al. 2014) and protein trafficking (Bremond et al. 2009; Choi et al. 1998; Sohn et al. 2001; Yoon et al. 2003). CD99 expression is indeed essential for the regulation of the transendothelial migration (TEM) of various immune cells including leukocytes (Watson et al. 2015), monocytes (Schenkel et al. 2002), neutrophils (Lou et al. 2007), and CD34+ cells (Imbert et al. 2006).

The regulatory role of CD99 have been implicated in pathological conditions. High CD99 expression has been observed in Ewing sarcoma (EWS) (Ambros et al. 1991; Llombart-Bosch et al. 2009), lymphoblastic lymphoma/leukemia (Dworzak et al. 2004), myeloid malignancies (Chung et al. 2017) and malignant gliomas (Seol et al. 2012; Urias et al. 2014) and sporadically in synovial sarcoma (Fisher 1998), mesenchymal chondrosarcoma (Brown and Boyle 2003), rhabdomyosarcoma (Ramani et al. 1993), thymic tumors, hemangiopericytoma (Rajaram et al. 2004), gastrointestinal and pulmonary neuroendocrine tumors (Goto et al. 2004), sex-cord stromal tumors (Baker et al. 1999) and a small percentage of breast carcinomas (Milanezi et al. 2001). However, there is an emerging group of neoplasms, including pancreatic endocrine neoplasms, gastric adenocarcinoma (Jung et al. 2002; Maitra et al. 2003) and osteosarcoma (OS) (Manara et al. 2006), in which CD99 expression is diffuse in benign diseases and absent in the malignant counterparts.

CD99 has been reported to have a marked effect on the migration, invasion and metastasis of tumor cells through multiple and still controversial mechanisms of action (see below for details), thereby emerging as a novel therapeutic target. CD99 had promising preclinical effectiveness in xenograft models, and thus paves the way for further development of antibodies to be used in clinical settings. Of note, CD99 engagement increases natural killer (NK) cell-mediated tumor lysis by inducing heat shock protein 70 (HSP70) expression (Husak and Dworzak 2012) and induces tumor cell death through non-conventional mechanisms, such as methuosis (Manara et al. 2016) or the induction of oncogenic stress (Chung et al. 2017; Husak and Dworzak 2017), as described for a number of oncogenes such as *RAS* (Serrano et al. 1997), *c-MYC* (Evan et al. 1992) and *BCR-ABL* (Dengler et al. 2011).

Thus, CD99 can be used as a robust marker for several tumors and a promising therapeutic target in cancer, particularly in tumors arising from the transformation of stem/precursors cells. Recently, CD99 was also shown as a unique marker of the epidermis, being strongly expressed in the basal/precursor cells of the epidermis and in hair follicles (Choi et al. 2016). Additionally, it was shown to participate in T cell recruitment into inflamed skin (Bixel et al. 2004), therefore appearing as a novel potential target for the treatment of dermatologic lesions.

Despite increasing evidence that CD99 has important functions in several aspects of cell biology, this molecule has been largely ignored by the scientific community, very likely because its functions have been confined to very specific areas of interest for many years. A number of unresolved issues remain to be clarified, particularly in terms of the mechanisms of action of CD99. This review discusses recent mechanistic studies that have had a major influence in the understanding of the role of CD99 in various aspects of physiology, cancer biology and therapeutics. No CD99 counterpart has been identified with certainty in mice considering the distant homologies of CD99-like molecules described in both humans and mice (Bixel et al. 2004). Thus, this review refers mostly to data on human CD99.

## Key Points

- *Together with Xga and CD99L2, CD99 constitutes a family of molecules that show no homology to any other known family*.
- *CD99 has two isoforms, a long form with 185 amino acids (known as CD99wt, CD99 type I, or CD99LF) and a short form with 161 amino acids (known as CD99sh, CD99 type II, or CD99SF) generated by alternative splicing of the cytoplasmic region*.
- *CD99 acts through homophilic interactions and enables homo- or heterotypic cell aggregation*.
- *CD99 can mediate diverse cellular processes such as adhesion, transendothelial migration, differentiation, and cell death, thereby affecting immune functions, inflammation and cancer metastasis*.
- *CD99 can act as an oncogene or as an oncosuppressor depending on the cellular context*.

## CD99 gene/protein structure

CD99 is encoded by the pseudoautosomal gene *MIC2*, which is located in the pseudoautosomal region (PAR) of both the X (Xp22.33-Xpter) and Y (Yp11-Ypter) chromosomes in humans (Aubrit et al. 1989; Banting et al. 1989; Ellis et al. 1994a, b; Fouchet et al. 2000; Goodfellow et al. 1986). The *MIC2* gene, localized to the proximal PAR1, is composed of 10 exons and is 50 kb in length.

To date, three CD99-related human genes which are the result of sequential duplications of an ancestral PAR during evolution have been described: a functional gene *PBDX* (pseudoautosomal boundary divided on the X chromosome) encoding the Xga antigen (Ellis et al. 1994a), the pseudogene *CD99L1* (CD99 antigen-like 1, also known as *MIC2*-related sequence (MIC2R)) and *CD99L2* (CD99 antigen-like 2) (Suh et al. 2003). In particular, *PBDX* codes for the Xga blood group antigen and shares a 48% homology with CD99 (Ellis et al. 1994b), while *MIC2R* (MIC2-related) is related to exons 1, 4, and 5 of *MIC2*. Transcripts from the *MIC2R* locus have been detected in all human tissues but none of them contains a functional open reading frame, making the role of *MIC2R* still unknown (Smith and Goodfellow 1994).

**Fig. 1:**
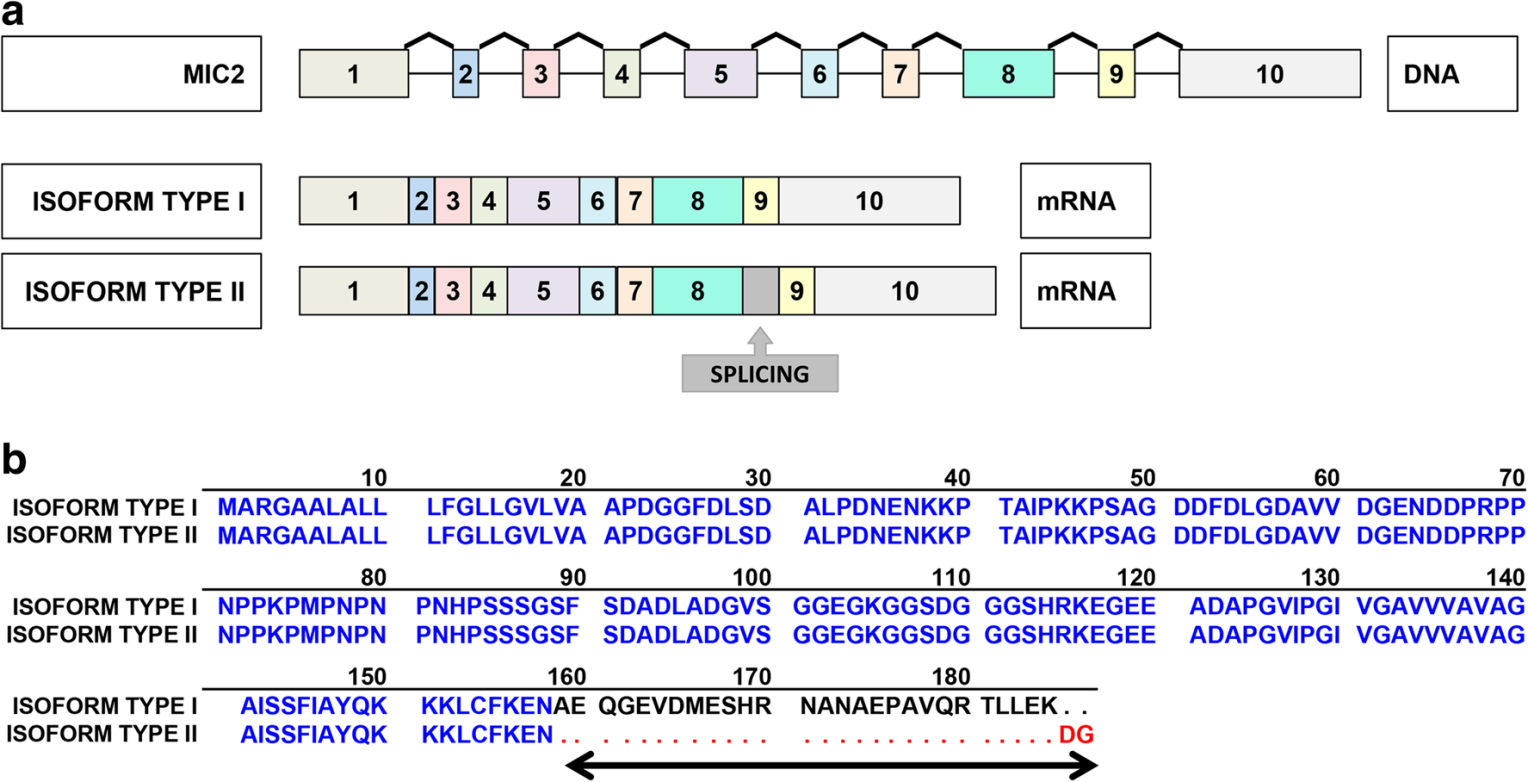
**a** Representation of the structural characteristic of the *MIC2* gene (DNA) and the two transcribed isoforms: CD99 type I and type II (mRNA). The splice site is indicated. **b** Amino acid sequences of CD99 isoform type I and isoform type II. The sequences of the two isoforms are aligned for comparison

The CD99 gene encodes two distinct proteins as result of alternative splicing process of the cytoplasmic region: a wild-type full-length CD99 or type I (CD99wt) with 185 aminoacids (corresponding to a molecular weight of 32 kDa) and a truncated form or CD99 type II (CD99sh) with 161 aminoacids (corresponding to a molecular weight of 28 kDa) (Hahn et al. 1997). The CD99sh transcript contains an 18-bp insertion at the boundary of exons 8 and 9 on the gene, which introduces an in-frame stop codon that generates truncated polypeptide (Hahn et al. 1997) (Fig. 1).

There are no predicted N-linked glycosylation sites, nor is there biochemical evidence for N-linked glycosylation of CD99 (Gelin et al. 1989); however, CD99 is extensively O-glycosylated, with carbohydrate chains accounting for 14 kDa (44%) of its apparent molecular size (Aubrit et al. 1989).

The CD99 protein is rich in proline residues and displays an organization typical of an integral membrane protein comprising an extracellular domain of 100 aminoacids, a transmembrane domain and a short intracellular C-terminal domain of 38 aminoacids. In addition, the cytoplasmic regions of the two CD99 isoforms contain shared motifs such as a zone rich in positively charged amino acids and a cysteine residue. An SHR motif for PKC and a leucine repeat are present only in the long form of CD99 (Mahiddine et al. 2016). It has been demonstrated that the cytoplasmic domain of the long form contains two putative phosphorylation sites, a serine at amino acid residue 168 (S168) and a threonine at amino acid residue 181 (T181). These potential phosphorylation sites may be important for intracellular signaling events and/or extracellular molecular interactions. In fact, the S168 of the long form of CD99 has been reported to be a site for PKC phosphorylation and it is required for the onco-suppressive function of CD99 in OS cells (Scotlandi et al. 2007).

The two isoforms of CD99 can naturally dimerize on the cell surfaces. The dimerization process begins in the Golgi apparatus and then the dimers are exported to the cell surfaces. At that point, CD99 acts as a receptor that becomes activated upon stimulation (Lee et al. 2008). The CD99 isoforms are expressed in a cell type-specific manner and dictate distinct CD99 functions (Alberti et al. 2002; Byun et al. 2006; Scotlandi et al. 2007). Specifically, the expression of the long form in a CD99-deficient Jurkat T cell line is sufficient to promote CD99-induced cell adhesion, whereas the co-expression of the two isoforms is required to trigger T cell death (Bernard et al. 1997). On B cells, the short form of CD99 inhibits homotypic adhesion, while the activation of the long form promotes cell–cell adhesion, indicating that the CD99 gene produces two distinct proteins with opposite functions regarding adhesion in lymphocytes (Hahn et al. 1997). In tumors, the two forms exert opposite effects on cell migration and metastasis (Byun et al. 2006; Scotlandi et al. 2007).

To determine the structural basis of CD99 functions, Kim and co-workers carried out structural studies on the cytoplamic domain of the long form of CD99 using circular dichroism and multi-dimensional NMR spectroscopy (Kim et al. 2004). The results revealed that the protein was unfolded and that it has a hairpin structure anchored by two flexible loops likely due to the heavy O-glycosylation of the CD99 protein. Consequently, human CD99 does not have any regular secondary structures (Kim et al. 2004).

Additionally, the search for CD99 homologs has been successful only in primates, indicating a high level of sequence divergence of this gene during evolution (Smith et al. 1993). Moreover, Park SH and collaborators reported the identification and characterization of a novel murine CD99 gene, known as D4, which was identified, as a mouse ortholog of the human CD99 according to phylogenetic analysis (Park et al. 2005). D4 is located in the C7-D1 region of chromosome 4. Genomic organization analysis revealed that the gene contains ten putative exons and the cDNA consists of nine exons that encoder a protein with 46% homology with human CD99 (Park et al. 2005). Rodent CD99 has a short cytoplasmic domain, resembling CD99 type II in humans. In contrast, putative bovine, porcine and chicken (*Gallus gallus*) CD99 genes are more similar to CD99 type I in humans, as is the cytoplasmic region of *Xenopus* CD99.

Suh YH and co-authors described the presence of a *CD99* paralogous mouse gene, *CD99L2* and its orthologs in human, rat, and zebrafish (Suh et al. 2003). The mouse *CD99L2* gene shares 45% homology with human *CD99* gene and 81% homology with the human *CD99L2 *gene. Rat CD99L2 shows 77% overall amino acid homology with mouse CD99L2 and 68% homology with human CD99L2, indicative of orthologous relationships among species. The deduced amino acid sequence of zebrafish CD99L2 shows 44%, 43%, 51% overall homology with mouse, rat, human CD99L2 respectively. Therefore, the *CD99L2 *genes in these species are all orthologous and evolutionarily conserved (Suh et al. 2003).

Regarding functions, only a partial overlap has been observed in roles and mechanisms between human CD99 and D4 or CD99L2. D4 has been identified as a ligand of the paired Ig-like type 2 receptor (PILR) (Shiratori et al. 2004). The cross-linking of D4 with PILR-Ig induces the apoptosis of thymocytes in the absence of T cell receptor signals constituting an active death signal, that removes thymocytes, predominantly at the double positive stage (Park et al. 2010) similar to some functions of human CD99 (see below).

CD99L2 is mainly expressed on leukocytes, endothelial cells and neutrophils (Bixel et al. 2007; Schenkel et al. 2007; Suh et al. 2003). Because of the high degree of conservation of five putative functional regions between mouse CD99L2 and human CD99, these two molecules may have similar functions (Liu et al. 2013). A majority of the studies on mouse CD99L2 have primarily described its roles in inflammation. Mouse CD99L2 is involved in the extravasation of neutrophils, monocytes, and T cells in mice under inflammatory conditions (Bixel et al. 2007, 2010; Schenkel et al. 2007; Seelige et al. 2013). In addition, the expression of CD99L2 on both leukocytes and endothelial cells suggest a possible role for this molecole in leukocyte–endothelial cell interactions during leukocyte extravasation, espacially during diapedesis (Schenkel et al. 2007).

## CD99 functions in normal cells

Although expressed in virtually any human cell types at low levels, CD99 is expressed at particularly high levels in specific cell types, including cortical thymocytes, pancreatic islet cells, ovarian granulosa cells, Sertoli cells of testes, endothelial cells, ependymal cells, bone marrow CD34+ cells, stromal lymphocytes and a broad range of hematopoietic cells, with the highest expression in the most immature lymphocytes and granulocytes, such as immature thymic T-lineage cells and tonsillar lymphoid progenitor cells (Banting et al. 1989; Dworzak et al. 1994; Edlund et al. 2012; Gelin et al. 1989; Levy et al. 1979; Tippett and Ellis 1998). Strong expression of CD99 has also been reported in immature basal keratinocytes (Choi et al. 2016).

**Fig. 2:**
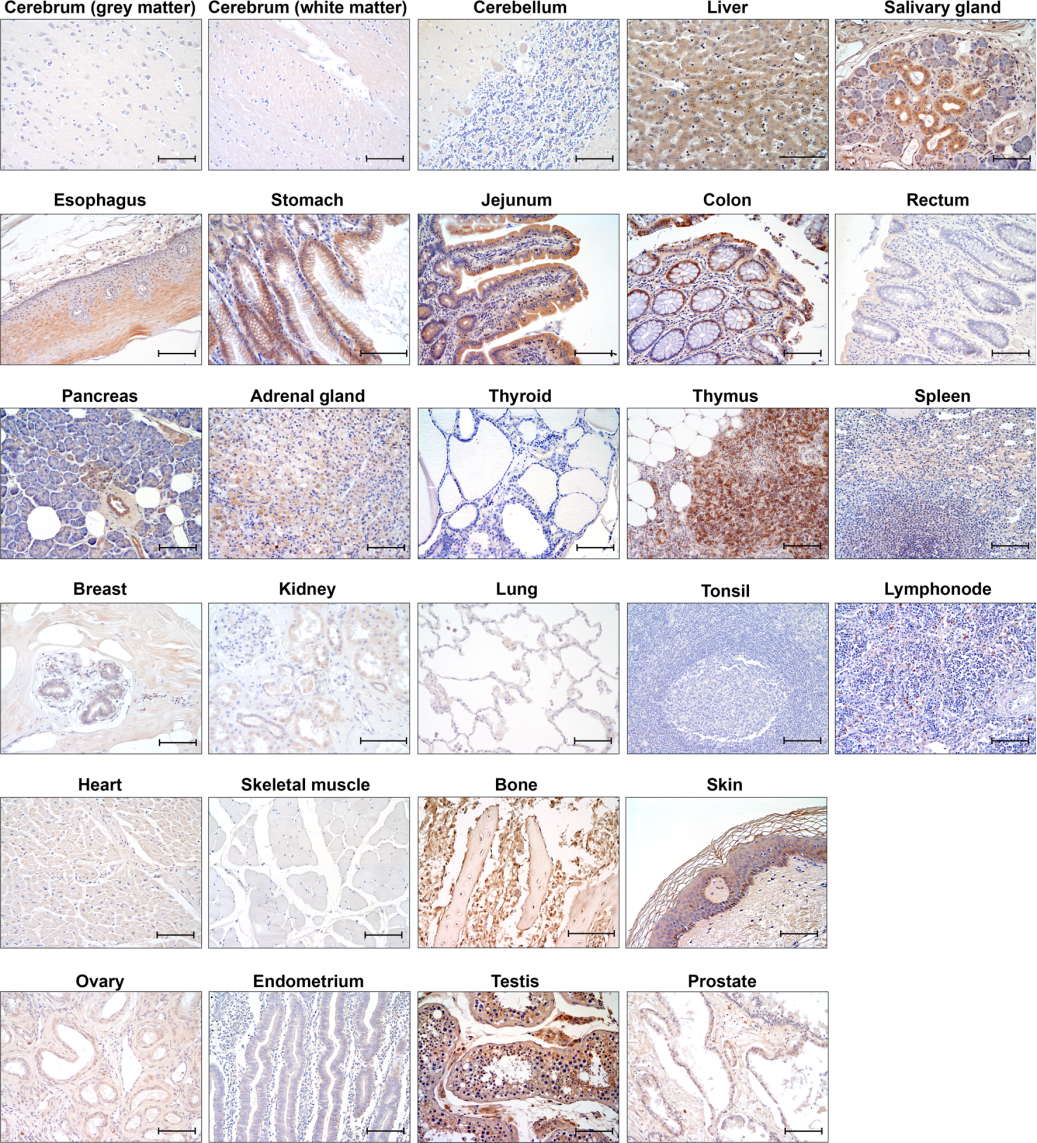
CD99 protein expression detected by immunohistochemical analysis in normal human tissue samples (scale bar: 100 μm was shown). Anti-CD99 primary antibody: O13 (Biolegend, cat.# 915,601; dilution 1/80)

Figure 2 shows examples of high and low expression of CD99 in different human tissues and cells.

CD99 was originally described as a molecule involved in the rosette formation of T cells with erythrocytes, indicative of its role as an adhesion molecule (Bernard et al. 1988).

Currently, CD99 is well known to play key roles in several biological processes such as: cell adhesion (Bernard et al. 1995, 2000; Cerisano et al. 2004; Hahn et al. 1997; Kasinrerk et al. 2000), apoptosis (Bernard et al. 1997; Cerisano et al. 2004; Husak et al. 2010; Jung et al. 2003; Pettersen et al. 2001; Sohn et al. 1998), T cells differentiation (Bernard et al. 1995, 1997), lymphocytes diapedesis to inflamed vascular endothelium (Dufour et al. 2008; Watson et al. 2015), and regulation of intracellular membrane protein trafficking (Bremond et al. 2009; Choi et al. 1998; Sohn et al. 2001; Yoon et al. 2003). Thus CD99 is important in peripheral immune responses and in processes including hematopoietic and neural precursor cell differentiation (Park et al. 1999).

Although the two CD99 isoforms have been reported to dictate distinct functional events (Alberti et al. 2002; Byun et al. 2006; Wingett et al. 1999) very few studies demonstrate how the two isoforms are expressed in appropriate cellular contexts and how they affect CD99-mediated intracellular pathways. For example, on T cells, CD99 can be expressed on the cell surface either as the long form (type I) or as heterodimers composed of the long form and the short form (type II) (Hahn et al. 1997). The heterodimeric variants are usually found on double-positive thymocytes and some immature T cell lines. However, single-positive thymocytes and peripheral T cells express the long form of CD99 only (Alberti et al. 2002).

Experiments involving CD99 transfection into CD99-deficient Jurkat T cells have demonstrated that both isoforms are required for the induction of apoptosis, whereas the presence of either isoform is sufficient to modulate cell adhesion; however the activation of the actin cytoskeleton requires the expression of the long isoform only (Alberti et al. 2002). When co-expressed, the two isoforms form covalently bound heterodimers, that localize within glycosphingolipid rafts and induce sphingomyelin degradation. Cholesterol depletion experiments have shown that this localization is required for the induction of apoptosis (Alberti et al. 2002). On B cells, the short form of CD99, inhibits homotypic adhesion, while the long form promotes cell-cell adhesion. The opposite effects of CD99 isoforms on homotypic B cell aggregation were shown result from their opposing functions in the regulation of the expression of the cell adhesion molecule LFA-1 (Hahn et al. 1997).

**Fig. 3:**
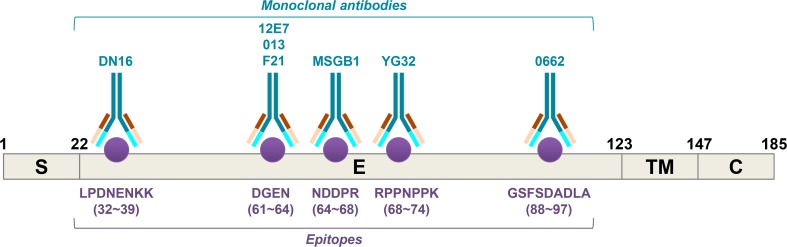
Schematic representation of the CD99 protein (S: signal sequence; E: extracellular domain; TM: transmembrane domain; C: cytoplasmic domain). The locations of the epitopes recognized by anti-CD99 mouse monoclonal antibodies are highlighted. The aminoacid sequence and the length of each epitope are also shown (Modified from Jung et al. 2003)

These data support the need of more extensive evaluation of the expression of CD99 isoforms in different cells and a deeper understanding of their role in physiology and pathology. However, most of the information available pertains to the long form of CD99, and this review specifically discusses these data. Most of the information reported in the literature has been obtained by the engagement of CD99 with murine monoclonal antibodies (mAbs) such as DN16, 12E7, O13, F21, MSGB1, YG32 and 0662 (see Fig. 3 for details) (Jung et al. 2003). Natural circulating ligands for human CD99 have not been described as of yet. CD99-mediated signaling activation is thought to arise from homophilic interactions among CD99 molecules on interacting cells (Schenkel et al. 2002) and this phenomenon further reinforces the importance of the level of expression of CD99 for determining its physiological functions.

### Role of CD99 in lymphocyte development and functions

CD99 is considered an important player in lymphocyte development. In studies on anencephaly, CD99-deficient fetuses typically demonstrated a marked impairment in thymic development, which suggests a role of CD99 in normal thymus ontogeny (Shin et al. 1999). In thymocytes, CD99 was shown to elicit homotypic cell aggregation as well as induce cell death at critical stages of thymocyte differentiation, when positive selection is known to occur (Bernard et al. 1995, 1997; Pettersen et al. 2001). In particular, CD99 mediates cell death of immature CD4 + CD8+ thymocytes that have an intermediate CD3 density, including all detectable CD69+ cells, however, CD99 has no effect on the survival of other thymocytes or T cells (Bernard et al. 1997). Consistently, CD99-induced cell death signaling occurs independently of major signaling pathways implicated in the control of thymocytes and mature peripheral T cells. CD99 engagement induces phosphatidylserine exposure on the surface of the immature thymocytes (Aussel et al. 1993; Choi et al. 1998), but cell death proceeds through classical or non-classical apoptotic pathways depending on the different CD99 domains that are activated by distinctive antibodies (Aussel et al. 1993; Pettersen et al. 2001). The reason behind why different CD99 domains are linked to different death pathways is not clear, but a similar complex situation in which the engagment of distinct domains induces either caspase-dependent or caspase-independent cell death has either been reported with other molecules such as the major histocompatibility complex (MHC) class I molecules (Genestier et al. 1998; Pettersen et al. 1998). Of note, CD99 participates to the upregulation of MHC class I and II and TCR expression on the thymocytes (Choi et al. 1998; Sohn et al. 2001). This increase is a result of accelerated mobilization of molecules stored in cytosolic compartments to the plasma membrane, rather than increased RNA and protein synthesis and it is more evident in the TCR-low subpopulations of immature double-positive thymocytes (Choi et al. 1998). By enhancing the efficiency of TCR-MHC interactions (Hahn et al. 2000; Waclavicek et al. 1998; Wingett et al. 1999), CD99 may create more opportunities for the positive selection of thymocytes. Thus, CD99 has a dual and contradictory function, and it participates in the cell maturation when cell death and selection occur also take place.

The engagement of MHC class II molecules has an antagonistic effect on CD99 engagement-related phenotypes (Kim et al. 2003), suggesting that complex regulatory interactions exist between CD99 and MHC class I and II signaling during thymocytes development and maturation (Kim et al. 2003). In addition, several lines of evidence have indicated a possible role of CD99 in T cell activation. Stimulation of CD99 with agonistic antibodies enhanced the expression of several T cell activation markers on anti-CD3-activating T cells and induced the elevation of intracellular Ca2+ and tyrosine phosphorylation of cellular proteins (Waclavicek et al. 1998; Wingett et al. 1999), leading to differential activation of mitogen-activated protein kinase (MAPK) members, including extracellular signal-regulated kinase (ERK), JNK and p38 MAPK and src kinase (Hahn et al. 2000; Lee et al. 2002). Binding of CD99 and suboptimal CD3-induced T cell activation led to translocation of TCR complexes to lipid rafts, without concomitant migration of CD99 to the rafts, or consequent enhancement of TCR-mediated signaling (Oh et al. 2007). Upon T cell activation, CD99 translocates to immunological synapses and anti-CD99 mAb has been shown to inhibit T cell proliferation, indicating an important role for CD99 in T cell activation (Pata et al. 2011). Moreover, CD99 is required for the effect of IFN-γ on HLA class I expression (Bremond et al. 2009).

CD99 may also have a pivotal role in early B lymphopoiesis. The levels of CD99 type I protein and mRNA have been found to be significantly linked to the maturation of normal B cell precursors (BCPs), with the highest levels observed in the most immature stage 1. The alternatively spliced CD99 type II mRNA is either absent in normal BCPs or present at extremely low levels with no effect on maturation (Husak et al. 2010). In these very immature normal BCPs, the binding of CD99 with corresponding mAb can induce cell death after long term incubations (7 days), suggesting a physiologic role of CD99 in clonal deletion necessary for B cell selection.

In B cell subsets from human tonsils, CD99 expression was found to be highest in tonsillar plasma cells (PCs). Furthermore, CD99 engagement did not influence apoptosis, differentiation, or antibody secretion of PCs but it reduced chemotactic migration of PCs toward CXCL12 and reduced ERK activation by CXCL12, suggesting that CD99-engaged PCs were less sensitive to the chemoattractive stimuli of CXCL12 (Gil et al. 2015).

### Role of CD99 in cell adhesion and leukocyte diapedesis

CD99 can act as an adhesion molecule and CD99 engagement has also demonstrated to induce the expression of adhesion molecules, including LFA-1, α4β1, ELAM-1, VCAM-1 and ICAM-1 (Alberti et al. 2002; Bernard et al. 2000; Dustin and Springer 1989; Hahn et al. 1997), which are associated with leukocyte adhesion and TEM, a critical step during the inflammatory processes. CD99 is expressed at the intercellular borders of endothelial cells (Schenkel et al. 2002) and it has been shown to be essential for TEM of monocytes, neutrophils, lymphocytes and CD34+ cells both *in vitro* (Lou et al. 2007; Manes and Pober 2011; Winger et al. 2016) and *in vivo* (Dufour et al. 2008; Imbert et al. 2006; Watson et al. 2015). CD99 has been shown to function downstream of PECAM, another critical molecule involved in TEM (Lou et al. 2007; Schenkel et al. 2002; Sullivan et al. 2013) and to act through homophilic interactions. Recently, the homophilic interaction of endothelial CD99 with leukocyte CD99 was shown to facilitate TEM of leukocytes through the activation of protein kinase A (PKA) via a signaling complex formed with the lysine-rich juxtamembrane cytoplasmic tail of CD99, the A-kinase anchoring protein ezrin, and soluble adenylyl cyclase (sAC) (Watson et al. 2015). PKA can then stimulate membrane trafficking from the lateral border recycling compartment to sites of TEM, thereby facilitating the passage of leukocytes across the endothelium. In these studies, blocking of CD99 by mAbs or by gene inactivation (Goswami et al. 2017) arrested migrating cells within the endothelium.

Additionally, migration of T cells into the skin (Bixel et al. 2004) and of neutrophils and monocytes into the peritoneal cavity (Dufour et al. 2008) can be blocked by interfering with CD99 functions, indicating the potential therapeutic applications of CD99 in the control of inflammation and immune cell infiltration. Recently, CD99 has been shown to be cleaved by meprin β, a multidomain type I transmembrane metalloprotease that acts as an initiator of regulated intramembrane proteolysis of cell adhesion molecules, has been demonstrated (Bedau et al. 2017). Meprin β cleaves CD99 at the cell surface and influences CD99-dependent permeability of endothelial cells.

### Role of CD99 in mesenchymal differentiation and osteoblastogenesis

CD99 is expressed in human mesenchymal stem cells at variable levels (Elsafadi et al. 2016; Rocchi et al. 2010; Sciandra et al. 2014). CD99 expression has been reported to decrease during the differentiation of mesenchymal stem cell toward a neural phenotype (Rocchi et al. 2010), while CD99 expression was shown to increase during normal osteoblastogenesis and osteoblast maturation (Sciandra et al. 2014). The CD99-encoding gene *MIC2* has been shown to be controlled by the transcription factor RUNX2 (Bertaux et al. 2005), which is essential for human osteoblast differentiation (Lian et al. 2004). CD99 was found in cell adhesion structures of osteoblastic cell cultures *in vitro*, and on osteoblasts adhering to one other and lining the bone surface in tissue samples *in vivo* (Manara et al. 2006). More recently, Oranger and colleagues have demonstrated an increase in CD99 levels during the differentiation of osteoblasts and bone marrow mononuclear cells, further supporting the role of CD99 in osteoblastogenesis (Oranger et al. 2015). The activation of CD99 with specific agonist antibody results in increased osteoblast differentiation and activation as demonstrated by the upregulation of alkaline phosphatase, Collagen I, RUNX2, and JUND expression.

## Role of CD99 in tumors

Although alterations in CD99 expression have been demonstrated in a broad range of neoplastic human tissues, the actual relationship of CD99 expression with the development of human cancers has been somewhat controversial, often with opposing functions, depending on the cellular context. High CD99 expression has been shown in EWS, and CD99 is routinely used for the differential diagnosis of EWS from other types of small round cell tumors in children (Ambros et al. 1991; Fellinger et al. 1991; Stevenson et al. 1994). CD99 knockdown in EWS cells transplated into immunodeficient mice induces terminal neural differentiation and reduces tumor growth, migration and bone metastasis (Kreppel et al. 2006; Rocchi et al. 2010), supporting a central role for CD99 in the pathogenesis of EWS. Several lines of evidence suggest a functional link between the aberrant transcription factor EWS-FLI, which is the pathogenetic driver of EWS, and CD99 (Hu-Lieskovan et al. 2005; Miyagawa et al. 2008; Riggi et al. 2008; Rorie et al. 2004). The oncogenic activity of EWS-FLI is facilitated by CD99, and consistently, EWS-FLI maintains high expression levels of CD99 (Hu-Lieskovan et al. 2005; Miyagawa et al. 2008; Rocchi et al. 2010) either directly through its binding to the CD99 promoter (Amaral et al. 2014; Rocchi et al. 2010) or indirectly through miRNA regulation (Franzetti et al. 2013). CD99 and EWS-FLI have opposite effects on EWS cell differentiation, while EWS-FLI induces neural differentiation, CD99 prevents it (Rocchi et al. 2010). The simultaneous expression of EWS-FLI and CD99 exerts a net effect on malignant cells to promote the expression of some neural features while maintaining cell growth capacity. Silencing CD99 in human EWS cell lines induces prolonged nuclear ERK1/2 phosphorylation (Rocchi et al. 2010), which seems to be crucial for shifting the biological functions of ERK1/2 toward neural development and differentiation (Cheng et al. 2013) reduces AKT and NF-κB signaling (Rocchi et al. 2010; Ventura et al. 2016) and orients the cells toward a terminal neural differentiation state regardless of the presence of EWS-FLI. Suppression of CD99 may thus serve as a mechanism to fine-tune the levels of transcriptional gene regulation, to shift the equilibrium in favor of cell differentiation rather than proliferation.

Apart from its role in EWS, CD99 was found to be frequently overexpressed in several types of leukemia, including acute lymphoblastic leukemia (ALL) (Dworzak et al. 2004), acute myeloid leukemia (AML) and stem cells in myelodysplastic syndromes (MDS) (Chung et al. 2017). CD99 appears to be a robust marker of cancer stem cells and a promising therapeutic target in these malignancies. Of note, the treatment of B and T cell leukemia lines with anti-CD99 antibody induces HSP70 expression, rendering these cells more prone to NK cell-mediated cytotoxicity (Husak and Dworzak 2012).

In pediatric B-cell leukemia/lymphoma, CD99 (*MIC2*) expression reflects maturation-associated patterns of normal B lymphopoiesis, with CD34+ cells expressing the highest levels of CD99 (Dworzak et al. 1999).

Regarding tumors of the central nervous system, a comprehensive study across a large series of astrocytomas of various grades has indicated a clear relationship between tumor aggressiveness and CD99 expression, with glioblastomas showing the highest positivity (Urias et al. 2014). In malignant gliomas, CD99 expression level is increased relative to that in normal tissues and is correlated with increased tumor aggressiveness and migration and invasion of tumor cells mediated by the Rho/Rac pathway (Seol et al. 2012).

In other tumors, such as Hodgkin lymphoma (Kim et al. 2000), OS (Manara et al. 2006; Scotlandi et al. 2007), prostate cancer and gastric cancer (Jung et al. 2002; Scotlandi et al. 2007), CD99 is expressed at low levels and the downregulation of CD99 rather than its over-expression seems to be required for tumorigenesis. The loss of CD99 expression is a significant molecular event for the induction of morphological and immunological phenotypes associated with Hodgkin’s and Reed-Sternberg cells (HRSCs) (Jian et al. 2015; Kim et al. 1998, 2000). These cells are more resistant to attack by cytotoxic T lymphocytes (CTLs) and apoptosis, indicating that the persistent lack of CD99 surface expression in HRSCs may favor their survival (Lee et al. 2011). In contrast, CD99 upregulation induces differentiation of Hodgkin lymphoma cells into terminal B-cells (Jian et al. 2015).

CD99 is known to function as a tumor suppressor in OS (Manara et al. 2006; Sciandra et al. 2014; Scotlandi et al. 2007; Zucchini et al. 2014). Forced expression of CD99 inhibits cancer metastasis through the suppression of C-SRC and ROCK2 activities (Scotlandi et al. 2007; Zucchini et al. 2014), while increasing osteoblast differentiation through ERK/RUNX2-mediated reactivation of osteoblastogenesis (Sciandra et al. 2014). CD99, which is expressed in osteoblasts, thus appears as a crucial regulator of malignancy in OS. Whenever tumor cells regain CD99 expression, they become prone to reactivation of terminal differentiation programs and lose migratory and metastatic propensities.

In gastric cancer, CD99 is present in normal gastric epithelium and its levels decrease in less differentiated tumors (Choi et al. 2004; Jung et al. 2002). In a group of 283 gastric adenocarcinoma samples, Lee JH et al. found that a decreased expression of CD99 was strongly associated with poor survival and unfavorable clinicopathological variables. The authors demonstrated that CD99 downregulation was due to hypermethylation of the protein-promoter region and loss of heterozygosity (LOH) of the CD99 gene locus together with SP1 downregulation (Lee et al. 2007). Similarly, among pancreatic tumors, CD99 is highly expressed only in pancreatic endocrine tumors (PETs), while the molecule is absent or weakly expressed in other histotypes (Goto et al. 2004). In gastrointestinal and pulmonary neuroendocrine tumors, there is an inverse correlation between CD99 expression and proliferation, local invasion and/or distant metastases (Pelosi et al. 2000; Pelosi et al. 2006).

Overall, CD99 is found to act either as an oncogene or as an oncosuppressor depending on the cellular context. Once the molecule is appropriately inhibited or induced, the net result is the reversion of tumor malignancy; these data establish CD99 as a promising therapeutic target for several tumors.

## CD99 and therapy

Despite being poorly studied, CD99 appears to govern many key components of cellular survival and metastatic processes and may have a relevant role in inflammation and cell adhesion processes. As indicated above, CD99 has indeed been reported to influence the following processes: 1. adhesion among different types of cells, either malignant or normal, well as to extracellular matrix (ECM) components; 2. extravasation across the endothelium; 3. cell survival, proliferation, differentiation and response to stress; 4. regulation of stem cell fate; 5. communication between immune cells as well as cancer cells and the tumor microenvironment via complex and still poorly defined molecular interactions.

**Fig. 4:**
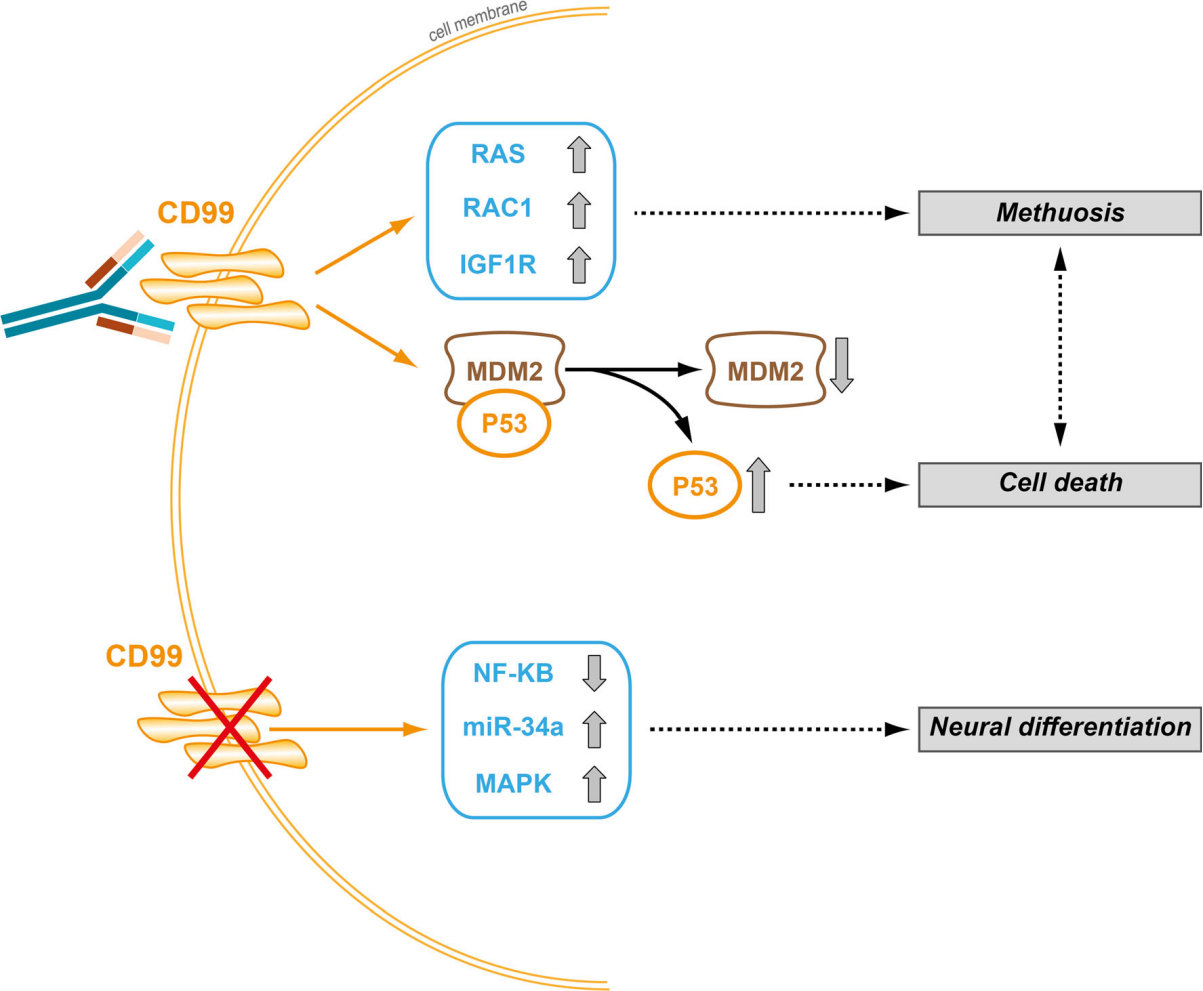
Schematic representation of CD99 signaling in EWS cells. The mechanistic relationships between CD99 silencing and neural differentiation or between antibody-mediated CD99 engagement and cell death are shown

Being a cell surface molecule, CD99 can be easily targeted by antibodies. Most of these antibodies have been reported to activate cell death signals and inhibit cell migration. This opens interesting therapeutic perspectives for tumors in which CD99 acts as on oncogene, such as EWS, ALL, MDS, AML and glioblastoma. CD99 engagement induces death in tumor cells via non-conventional, caspase-independent programmed cell death or through a non-apoptotic pathway resembling methuosis (Cerisano et al. 2004; Manara et al. 2016; Scotlandi et al. 2000; Sohn et al. 1998), a process characterized by excessive accumulation of vacuoles in the cytoplasm, leading to compromised cell viability (Maltese and Overmeyer 2014). The capability to induce non-conventional apoptotic signaling may be clinically relevant as tumor cells are generally resistant to classical apoptotic cell death. Accordingly, anti-CD99 antibodies exert additive/synergistic effects when combined with conventional agents, such as doxorubicin or vincristine (Guerzoni et al. 2015; Scotlandi et al. 2006) and are effective even against chemoresistant tumor cells (Manara et al. 2016). In EWS cells, antibody-mediated engagement of CD99 rapidly evokes caveolin-1-dependent endocytosis and promotes the upregulation of IGF-1R and RAS/Rac1 signaling, leading to defective vacuolization and death by methuosis (Manara et al. 2016). The effects are more dramatic in malignant cells that express high levels of CD99 and are facilitated by the reactivation of p53, resulting from the CD99-induced degradation of MDM2 (Guerzoni et al. 2015). Neither p53 reactivation nor RAS induction can be triggered in normal cells. In general, data obtained in both EWS and leukemia (Husak et al. 2010) indicate that CD99-induced cell death occurs preferentially in cells with an aberrant genetic background, thus conferring selectivity of anti-CD99 approaches against tumor cells. Figure 4 summarizes CD99 signaling in EWS cells. In addition, CD99 engagement increases NK cell-mediated tumor lysis by inducing HSP70 expression (Husak and Dworzak 2012) and inhibits tumor cell migration and metastasis through different mechanisms, including the suppression of C-SRC and ROCK2 activities (Pinca et al. 2017; Scotlandi et al. 2007; Zucchini et al. 2014) or the blockade of CD98-mediated β1 integrin signaling, which can suppress tumor progression by inhibiting the positive feedback loop involving CD98/β1 integrin/focal adhesion kinase (FAK)/RHOA/ROCK (Lee et al. 2017).

Therefore, mAbs against CD99 have promising preclinical effectiveness in several types of tumors (EWS, AML, ALL and glioblastoma) and are selective for malignant stem cells. Of note, a human diabody has been recently developed (the diabody binding site falls, very likely, into the CD99 extracellular domain included between residues 50 and 74) with *in vivo* efficacy against EWS (Gellini et al. 2013; Guerzoni et al. 2015; Moricoli et al. 2016), thus paving the way for further development of this approach.

## Critical issues/open questions

1. Due to the lack of sufficient reports on the different roles for the two CD99 isoforms, a more detailed study of their expression in normal and diseased cells is necessary.
2. The mechanisms of action of CD99 isoforms are strictly dependent on the cellular context and need further investigation.
3. CD99 likely acts by interacting with other cell surface molecules, which are responsible for its signaling. Hovewer, very limited data are available on the heterophilic interactions of CD99.
4. The functional relationship between human and murine CD99 is unclear.

## Conclusion

CD99 has been largely ignored by the scientific community. However, increasing evidence highlights a crucial role for this peculiar transmembrane molecule in cancer and inflammation. CD99 regulates fundamental biological processes and may have has promising clinical application in several fields of medicine. Efforts should thus be made to improve our understanding of the mechanisms of action of CD99, as it is time to give proper attention to this molecule.
